# Gastrointestinal Helminth Infections in Dogs in Sheep and Goat Farms in Greece: Prevalence, Involvement of Wild Canid Predators and Use of Anthelmintics

**DOI:** 10.3390/ani14223233

**Published:** 2024-11-12

**Authors:** Eleni I. Katsarou, Konstantinos V. Arsenopoulos, Charalambia K. Michael, Daphne T. Lianou, Efthymia Petinaki, Elias Papadopoulos, George C. Fthenakis

**Affiliations:** 1 Veterinary Faculty, University of Thessaly, 43100 Karditsa, Greece; 2 Laboratory of Parasitology and Parasitic Diseases, School of Veterinary Medicine, Faculty of Health Sciences, Aristotle University of Thessaloniki, 54124 Thessaloniki, Greeceeliaspap@vet.auth.gr (E.P.); 3 School of Veterinary Medicine, European University of Cyprus, Engomi 2404, Cyprus; 4 University Hospital of Larissa, 41110 Larissa, Greece

**Keywords:** anthelmintic treatment, canid predator, farm dog, hookworm, parasite, praziquantel, *Toxocara canis*, wolf

## Abstract

The objectives of the present work were the investigation of gastrointestinal parasitic infections in dogs in small ruminant farms in Greece, the elucidation of potential predictors for these infections and the description of practices related to administration of anthelmintics to dogs. This study was carried out in 444 small ruminant farms in Greece. Faecal samples were collected directly from the rectum of the dogs in the farms. The samples were processed by means of conventional parasitological techniques, specifically, a combined sedimentation flotation technique. There were dogs in 92.8% of the farms, with a median number of four dogs per farm. The following variables were associated with the presence of dogs in the farms: the presence of wild mammal predators near the farms, the increased daily period of farmers’ presence at the farm, goats as the livestock species at the farm and the management system applied in the farm. Helminth eggs were detected in samples from 72.6% of the farms. The main helminth eggs detected were those of hookworms (*Uncinaria*/*Ancylostoma*) and *Toxocara canis*, in 68.6% and 51.3% of the farms, respectively. In our multivariable analyses, an association emerged between the presence of canid predators near a farm and the detection of these helminths in faecal samples: in 76% and 60% of the samples, respectively, versus in 58% and 39% of the samples from farms with no canid presence. Of farmers with dogs, 16.0% reported that they omitted the administration of anthelmintics to the animals. In multivariable analysis, the semi-extensive or extensive management system applied in the farm, the lower annual milk production per animal and the lack of collaboration with a veterinary practice were the significant predictors for the omission of anthelmintic administration to the farm dogs. There was also a clear association in the omission of anthelmintic administration to the dogs and to the livestock on the farm. The most frequently administered anthelmintic was praziquantel, which was used in 93.6% of the farms.

## Simple Summary

This study explored facets of gastrointestinal parasitism in dogs in small ruminant farms in Greece. Helminths were detected in faecal samples of the dogs in 73% of the farms. Hookworms and roundworms were found most frequently, specifically in the faecal samples of dogs in 69% and 51% of the farms. There was an association between the detection of these helminths in faecal samples from dogs and the presence of canid predators near a farm. A small proportion of farmers (16.0%) reported that they omitted the administration of anthelmintics to these animals. The variables found to be significant for this omission were the semi-extensive or extensive management system applied in the farm, the lower annual milk production per livestock animal and the lack of collaboration with veterinary practice.

## 1. Introduction

Dogs have been serving an important role in farming businesses for very many years as guardians and herders of livestock. Their importance is particularly true for small ruminant farms, as these animals are frequently attacked by wildlife predators, against which farm dogs can offer significant protection [1]. Indeed, relevant surveys have shown that a main reason for owning farm dogs is the protection of livestock from predators [2,3]. Further, farm dogs can provide security against various other unwanted actions against the farm (e.g., theft).

Farm dogs can also be sources of various pathogens for transmission to sheep or goats directly or indirectly. These include viruses (e.g., *Rabies virus* [4]), bacteria (e.g., *Leptospira* spp. [5] and *Anaplasma ovis* [6]), protozoa (e.g., *Babesia ovis* [7]) and metazoan parasites (e.g., *Taenia multiceps* [8]). Additionally, farm dogs might disseminate zoonotic pathogens to farmers and farm staff, which has an impact on public health (e.g., *Leptospira* spp. and *Echinococcus granulosus*).

With regard to parasites, sheep and goats are intermediate hosts for some of the gastrointestinal helminths of dogs. For *Echinococcus granulosus*, the hydatid cyst is the larval form that develops in sheep and goat tissues (mainly in the lungs and the liver); for *Taenia multiceps*, the *Coenurus cerebralis* is the larval form that develops in the nervous tissue of sheep and goats; for *Taenia ovis*, the *Cysticercus ovis* is the larval form that develops in the muscles of sheep and goats; and for *Taenia hydatigena*, the *Cysticercus tenuicollis* is the larval form that develops in the viscera of sheep and goats. Practices that have been reported to increase the risk of infection with these helminths in farm dogs, and thus the development of the respective larval stages in sheep and goats, include the consumption of the offal of dead small ruminants by farm dogs (either through the direct offering of offal to dogs or consequently to the improper disposal of carcasses) and the omission of anthelmintic treatments in farm dogs [9]. Occasionally, livestock can be paratenic hosts (i.e., hosts in which parasite development does not occur, but these can serve to bridge an ecological or trophic gap in a parasite’s life cycle [10]). A case of larval toxocarosis was reported in sheep after oral challenge with 10,000 *Toxocara canis* eggs, with histological changes present in the small intestine, the liver and the lungs [11].

The application of appropriate preventive measures in farm dogs (e.g., vaccinations and anthelmintic treatments) help to control the dissemination of the above pathogens to livestock, other dogs and humans in contact with the farm dogs.

The objectives of the present work were the investigation of the gastrointestinal parasitic infections in dogs in small ruminant farms (hereafter referred to as ‘farm dogs’) in Greece, the elucidation of potential predictors for the infections and the description of practices related to the administration of anthelmintics to dogs. This work was carried out as part of a large field study in sheep and goat farms throughout Greece.

## 2. Materials and Methods

### 2.1. Visits to Farms and Sample Collection

**Figure 1:**
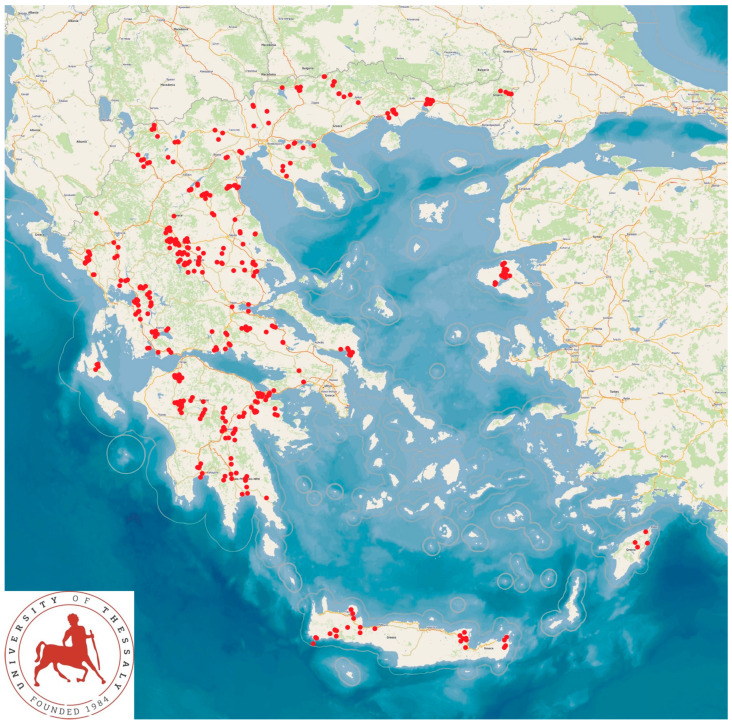
Locations of the 444 small ruminant farms across Greece, wherein gastrointestinal parasitic infections of farm dogs were studied.

This study was carried out in 444 small ruminant farms in Greece (325 sheep and 119 goat farms) from April 2019 to July 2020. The farms were located throughout the country (Figure 1). The farms were enrolled into this work on convenience basis, i.e., the willingness of respective farmers to receive a visit by university personnel for collecting samples from animals and for obtaining information. All the farms were visited by the authors. The details of these procedures have been presented previously by Lianou et al. [12].

During the visit to the farms, first, the dogs on the farms were counted. Then, faecal samples were collected. Specifically, samples were collected from dogs in 226 farms; (i) the farmer consented to faecal sampling of the dogs and (ii) the dogs had not received anthelmintic treatment for three months prior to the visit. For the sampling, the dogs were restrained, and faecal material (3–5 g) was collected directly from the rectum. For sampling, the gloved finger of the investigator was inserted into the animal’s rectum and a small quantity of faecal material was obtained and withdrawn. The glove was then used as a ‘bag’ to maintain the specimen.

Samples were collected from all the animals on the premises, specifically one sample was obtained from each dog. In 28 farms, it was not possible to collect samples from some of the farm dogs (1–3), due to (i) the difficulty in rounding up the animals, (ii) aggressiveness of the dogs or (iii) lack of faecal material in the rectum.

An interview was carried out with the farmer, always by the same researcher (author D.T.L.), by means of a structured questionnaire [12], in order to obtain information regarding the farm.

The samples were collected into numbered plastic bags corresponding to the individual animals. The samples were transported to the laboratory by the researchers and stored at temperatures from 8.0 to 10.0 °C using portable refrigerators.

### 2.2. Laboratory Examinations

Parasitological examinations started within 3 days after sample collection. Initially, faecal material from all dogs at the same farm was pooled, as the farm was the unit of reference. Pooled material was formed by obtaining a quantity of 2 g of faeces from each individual dog and thorough mixing and homogenisation (which served to release eggs from proglottids) in a blender, for processing.

Established parasitological techniques were employed in the pooled faecal material. Specifically, a combined sedimentation flotation (zinc sulphate 33.2%, specific gravity 1.3) technique was applied [13,14]. Testing was applied in triplicate in each pooled faecal material. Differentiation of the eggs of *Toxocara canis* and *Toxascaris leonina* was performed based on their morphological characteristics.

### 2.3. Data Management and Analysis

The presence of gastrointestinal helminths in the dogs on a farm was confirmed when the corresponding eggs were detected in at least one of the three samples processed from the pooled faecal material of the respective farms.

During the visits to each farm, data on farm location were collected using hand-held Global Positioning System Garmin units. The geo-references were resolved to specific farm levels. Climatic variables prevailing at the location of each farm were subsequently derived from ‘The POWER (Prediction of Worldwide Energy Resources) Project’ (NASA Langley Research Center (LaRC), Hampton, VA, USA), which provides meteorological datasets from NASA research for the support of agricultural needs. The following settings were used for obtaining the data: user community: ‘agroclimatology’; temporal average: ‘daily & annual’; latitude/longitude: ‘geo-references of each farm’; output file format: ‘*ASCII*’. Subsequently, data for climatic parameters were extracted [15]. For the evaluation of variables related to climatic conditions at the locations of the farms, data three months and the year preceding the visit were taken into account.

All the data were systematically recorded and organised using Microsoft Excel. Initially, basic descriptive analyses were performed, and exact binomial confidence intervals (CIs) were obtained. The frequency of the various outcomes was compared in tables of cross-categorised frequency data by the use of Pearson’s chi-square test or Fisher’s exact test as appropriate. Comparisons between continuous data were performed by use of the Mann–Whitney test or the Kruskal–Wallis test as appropriate. Correlations were assessed by means of Spearman’s rank correlation.

The following outcomes were considered: (i) ‘presence of farm dogs in small ruminant farms’, (ii) ‘detection of hookworms in faecal samples from a farm’, (iii) ‘detection of *Toxocara canis* in faecal samples from a farm’ and (iv) ‘omission of the administration of anthelmintics to farm dogs’. In total, 16 parameters for outcomes (i) and (iv) and 34 parameters for outcomes (ii) and (iii) among the information obtained collected during this study (Table S1) were evaluated for potential association with these outcomes in univariable analyses. Then, multivariable analyses were performed using mixed-effects logistic regression with farms as the random effect and initially offering to the respective model all variables, which achieved a significance of *p* < 0.2 in the univariable analysis and progressive removal of variables. The variables included in the final multivariable models constructed are detailed in Table S2.

The omission of the administration of anthelmintics to farm dogs and four more significant variables for that outcome were evaluated using a principal component analysis.

Statistical significance was defined as *p* < 0.05.

## 3. Results

### 3.1. Presence of Dogs in Small Ruminant Farms

There were farm dogs in 412 farms (92.8% (95% CIs: 90.0–94.9%)); in total, 2296 dogs were in these farms. The median number of farm dogs was 4 (interquartile range: 4) per farm.

There was a tendency for the presence of farm dogs more often in goat than in sheep farms: in 96.6% and 91.4% of the respective farms (*p* = 0.06). Also, the median number of farm dogs was significantly higher in goat than in sheep farms: 5 (5.5) versus 4 (4.0) animals per farm, respectively (*p* = 0.001).

**Figure 2:**
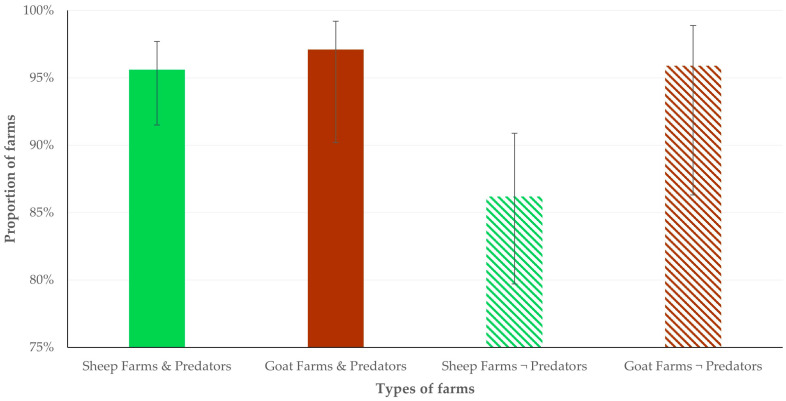
The proportions of small ruminant farms with the presence of farm dogs, in accord with the livestock species on the farm ^1^ and the presence of wild mammal predators near the farms ^2^ (bars indicate 95% CIs). ^1^ Sheep farms: green colour, goat farms: brown colour; ^2^ Predators: presence (full pattern) or no presence (motif pattern).

The results of the univariable analyses for the presence of dogs in the small ruminant farms are in Table S3. In the multivariable analysis for the presence of farm dogs, significant associations were found with the following variables: (a) the presence of wild mammal predators (grey wolves, golden jackals or brown bears) near the farms (*p* = 0.008), (b) the increased daily period of farmers’ presence at the farm (*p* = 0.021), (c) goats as livestock species on the farm (*p* = 0.044) and (d) the management system applied in the farm (*p* = 0.046) (Table 1, Figure 2).

**Figure 3:**
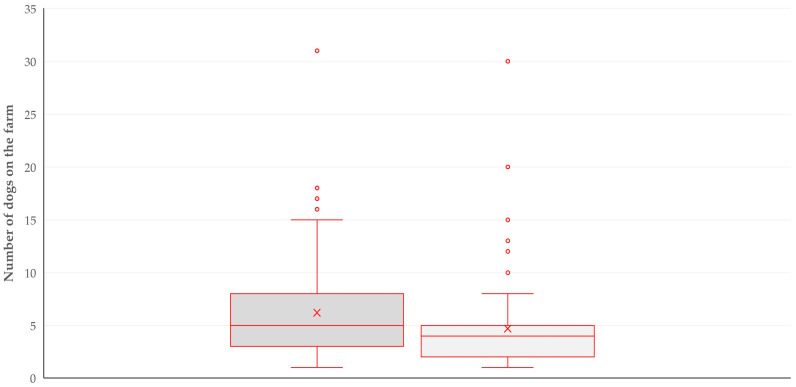
Box and whisker plot of the numbers of farm dogs on small ruminant farms in Greece, in accord with the presence (dark grey) or absence (light grey) of wild mammal predators near the farms.

For three of the above variables, there was also a significant association with the number of dogs on the farm. Specifically, a significant association was found with the presence of wild mammal predators near the farms: 5 (5.0) versus 4 (3.0) in farms with no predator presence (*p* < 0.0001) (Figure 3), the increased daily farmers’ presence at the farm (*p* = 0.002) and goats as the livestock species on the farm (*p* = 0.008).

### 3.2. Results of Parasitological Examination of Faecal Samples from Dogs

Helminth eggs were detected in the faecal samples from farm dogs in 164 farms (72.6% (95% CI: 66.4–78.0%)) (Figure S1). Of these, in samples from 114 farms (69.5%), the eggs of at least two helminths were detected. The frequency of detection of the various helminth eggs in the samples is in Table 2.

**Figure 4:**
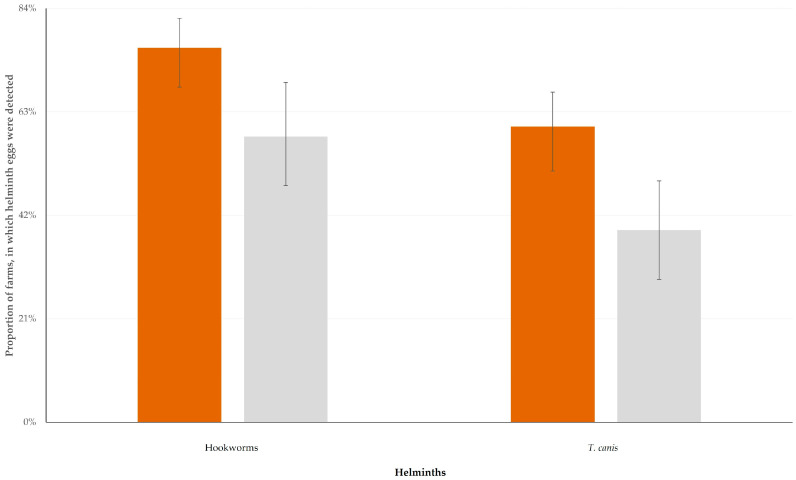
Proportions of small ruminant farms, in which eggs of hookworms or of *T. canis* were detected in faecal samples from farm dogs, in accord with presence (brown) or absence (grey) of canid predators near farms.

The results of the univariable analyses for predictors for the detection of the eggs of hookworms or *T. canis* in the faecal samples from the farm dogs are in Tables S4 and S5. In the multivariable analyses, the presence of wild canid predators near the farms emerged as significant predictor for the detection of hookworms and *T. canis* (*p* < 0.010) (Figure 4); further, the professional status of the farmers (full-time farming) had an association with the detection of hookworms (*p* = 0.042) as well as a tendency for association with the detection of *T. canis* (*p* = 0.07) (Table 3 and Table 4).

### 3.3. Administration of Anthelmintic Treatments in Dogs

#### 3.3.1. Anthelmintic Treatments in Dogs

**Figure 5:**
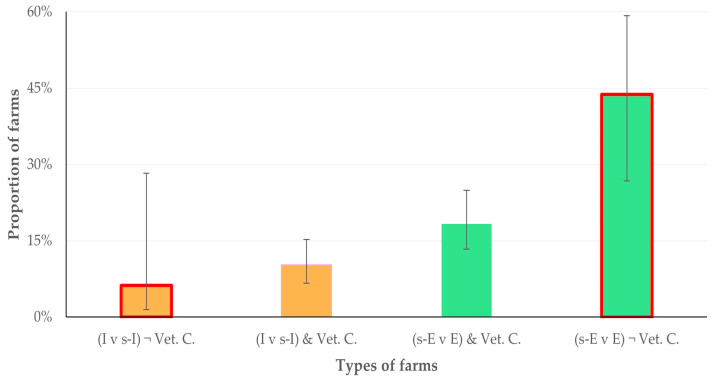
The proportions of small ruminant farms, in which anthelmintic treatments in the farm dogs were omitted, in accord with the management system applied in the farms ^1^ and the collaboration with a veterinary practice ^2^ (bars indicate 95% CIs). ^1^ I: intensive or s-I: semi-intensive (brown), s-E semi-extensive or E: extensive (green); ^2^ Vet. C.: collaboration with a veterinary practice, yes (no outline) or no (red outline).

**Figure 6:**
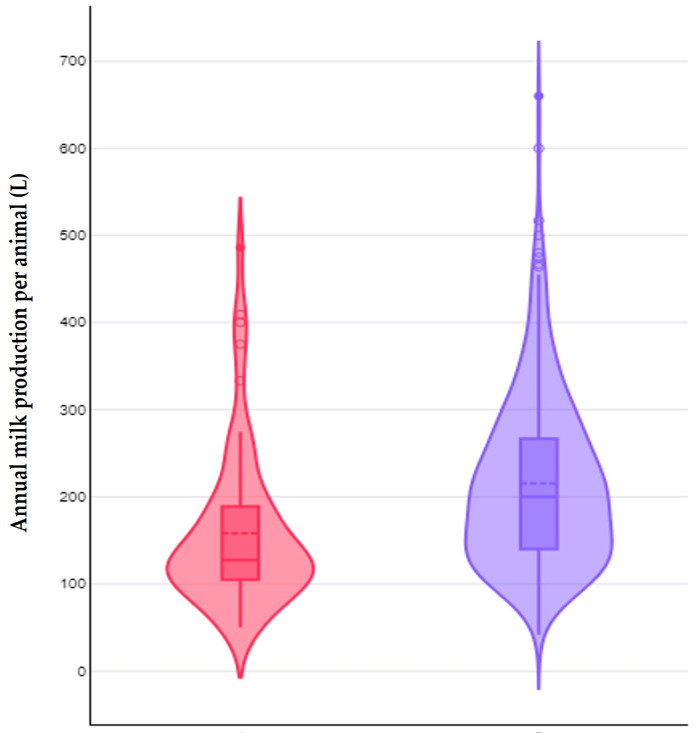
Violin plot of the annual milk production per animal in small ruminant farms, in accord with the omission (red) or performance (blue) of the administration of anthelmintics to the farm dogs.

**Figure 7:**
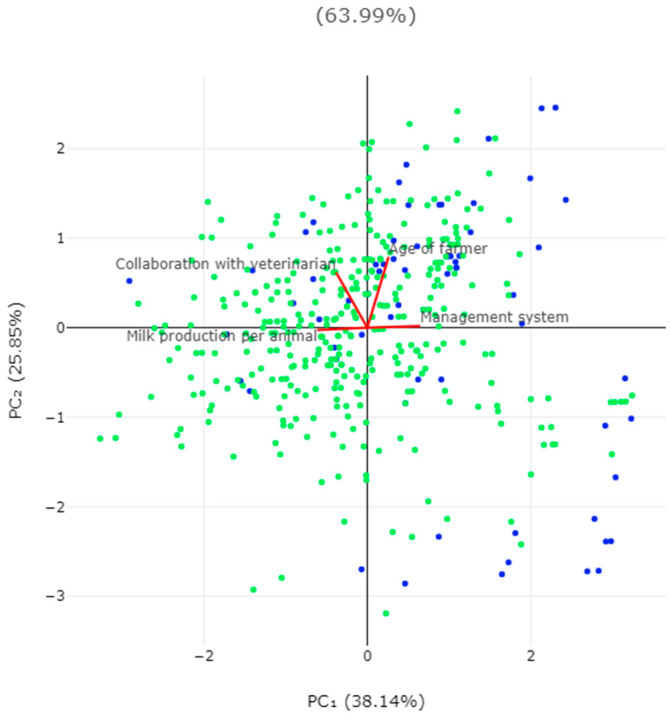
Bi-plot of results of principal component analysis for omission (blue dots) and performance (green dots) of administration of anthelmintics to farm dogs.

Among the farmers with farm dogs, 66 (16.0%) reported that they omitted the administration of anthelmintics to these animals. The results of the univariable analyses for predictors for the omission of anthelmintics to the farm dogs are in Table S6. In the multivariable analysis, the following variables emerged with a significant association with the omission of anthelmintic administration to the farm dogs: (a) the semi-extensive or extensive management system applied in the farm (*p* = 0.004), (b) the lower annual milk production per animal (*p* = 0.004) and (c) the lack of collaboration with a veterinary practice (*p* = 0.032) (Table 5, Figure 5 and Figure 6). There was also a tendency for association of the outcome with a higher age of the farmers (*p* = 0.06) (Figure S2). The above four variables explained 64.0% of the variation for omitting or performing the administration of anthelmintics to the farm dogs (Figure 7 and Figure S2, Table 6).

The most frequently used anthelmintic in the farm dogs was praziquantel (*n* = 324 farms; 93.6%). Details of the anthelmintic drugs given to the farm dogs are in Table 7.

With regard to specific pharmaceutical products, in 327 farms (94.5%), products with endoparasitic drugs exclusively were administered. In 19 farms, a product including a combination of endo- and ecto-parasiticides was used.

#### 3.3.2. Associations Between Anthelmintic Treatments in Dogs and Livestock Health

There was a clear association between the omission of anthelmintic administration to the farm dogs and to the livestock (sheep or goats) on the farms. Of the 66 farmers who did not administer anthelmintics to their farm dogs, 3 (4.5%) also reported that they also did not carry out such treatment in their livestock; in contrast, only 2 of the 346 (0.6%) farmers who gave anthelmintics to the dogs reported that they did not carry out such treatment in their sheep/goats (*p* = 0.007). Further, there was also an association in the use of ivermectin in farm dogs and livestock on the same farm. Of the 28 farms on which ivermectin was administered to dogs, in 26 (92.9%), it was also used as an antiparasitic in livestock (*p* < 0.0001) (Table S7).

Moreover, the proportion of farmers who declared coenurosis as an important health problem in the replacement livestock in their farms was significantly higher among those who omitted anthelmintic treatments in the farm dogs: 20.0% versus 10.7% among farmers who administered anthelmintics to dogs (*p* = 0.035).

## 4. Discussion

### 4.1. Presence of Dogs in Small Ruminant Farms

Dogs constitute a significant component in small ruminant farms. The use of farm dogs first appeared hundreds of years ago, with the aim to help farmers in the protection of their animals from predator wildlife animals [16]. The more frequent presence of farm dogs where shepherds and goatherds spend more time at the farm aligns with the complex ethno-ethological relationship between the farmers and the dogs [17]. In Greece, the ‘Greek shepherd dogs’, a breed descending from the ancient-breed Molosser-type sheep dogs, live well with the livestock and also can protect effectively against predation threats [18,19].

The present results corroborate that dogs play a clear and particular role in the protection of flocks and herds against wild mammal predators. This was confirmed by the more frequent presence of dogs and their higher numbers in farms near which the presence of wild mammal predators was also reported. Goats are managed more frequently under the semi-extensive or extensive management system, and usually, they would browse individually among bushes and scrubs, which may facilitate attacks by predators [20]. Sheep, in contrast, are managed more often under the intensive or semi-intensive system [21] and usually graze in open terrain spaces, which makes attacks by predators more difficult. The above are in accord with the more frequent presence of dogs on goat farms, as well as with their higher numbers in these farms. In Greece, Arcturos, a non-profit, non-government organisation focussing on the protection of wildlife fauna and natural habitat, engages in breeding and distributing puppies of farm dogs for free to livestock farmers in Greece [22].

The presence of farm dogs, coupled with other biosecurity measures (e.g., installation of fencing and housing livestock at night) can contribute to minimising livestock deaths from predation. The presence of farm dogs can lead to behaviour changes on the part of predators, which facilitate the co-existence of these animals with livestock and prevent attacks on sheep and goats [1]. In turn, this would lead to reducing human–wildlife conflict, which otherwise may lead to action on the part of farmers, e.g., hunting; for example, studies in Argentina and South Africa have indicated that the introduction of high numbers of dogs in farms has led to decreases in predation losses and increased tolerance to wild predators by affected farmers by over 75% [23,24]. In Greece, this is part of Arcturos’ work, which aims to mitigate human–wildlife conflicts by promoting the use of farm dogs on livestock farms in Greece [22].

### 4.2. Parasites in Faecal Samples from Dogs

Canine parasitic infections compromise the health and welfare of farm dogs. Dogs are also the definitive hosts of parasites, which can cause important disorders to sheep and goats, reducing financial outputs from farms. Also, these helminths may pose a zoonotic risk.

Hookworms, found to be the most common helminth in farm dogs, have a direct biological life cycle, which contributes to easy transmission of the worms, thus explaining the finding of canid predators as significant predictors for this infection. These parasitic species *(Ancylostoma caninum* and *Uncinaria stenocephala*) and *Toxoxara canis* are also parasites of canid predators [25,26,27]. Hence, the presence of canid predators around the livestock farms contributes to contamination of the farm environment with infective parasitic forms, which subsequently lead to the infection of the farm dogs. The canid predators can be a source of infection, through which a sylvatic cycle of infection for the gastrointestinal helminths is developed and maintained.

There was a difference in the frequency of the various helminths found in the present study compared to the results of the presence of gastrointestinal helminths in an urban population of dogs in Greece [28]. That study referred to *T. canis* as the most frequently detected helminth, whilst hookworms ranked second. This difference may possibly reflect infections of farm dogs from canid mammals as discussed above.

In addition to potential adverse effects to livestock, the zoonotic importance of the gastrointestinal helminths is also noted. An increased number of dogs (estimated to around 60,000 to 70,000) in Greece originated from farm dogs, which had escaped or were even abandoned [28,29]. Some of these animals might have moved into urban or peri-urban areas [29], which might increase the risk for transmission of these helminths to domestic dogs, thus transferring parasites from the sylvatic cycle into an urban one and increasing the risk for transmission to people.

### 4.3. Anthelmintic Treatments in Farm Dogs

The predictors for the omission of administration of anthelmintics to farm dogs reflect situations present in small ruminant farms. The association with farms under a semi-extensive or extensive management system reflects a possible difficulty in purchasing pharmaceutical products, as such farms are usually in mountainous areas, away from cities and major towns. The association with a lower average milk production by sheep and goats can be explained as the result of a smaller income available to those farmers to spend on anthelmintics for their dogs. The lack of association with veterinary practice reflects the reduced monitoring of animals on the farm and the erratic implementation of schemes for the control of animal diseases. Finally, the tendency for association with the increased age of farmers is in full accord with similar findings from other countries; for example, it has been reported from New Zealand that farmers older than 50 years routinely employ fewer health management tools and often omit procedures as basic as anti-clostridial vaccinations [30].

The anthelmintic drugs reported to be administered in the farm dogs reflect the pharmaceutical products licenced for use in dogs in Greece. A notable exception is the administration of ivermectin, which had not been licenced for dogs. The drug is frequently used in sheep and goats in Greece [21]; hence, its use in dogs seems to be a matter of convenience, given that the use of ivermectin in dogs and livestock on the same farm has been found. Ivermectin may potentially lead to safety issues in certain dog breeds [31,32]; therefore, its use in those animals must be avoided.

The omission of administration of anthelmintics to farm dogs can lead to health problems in the livestock of that farm. An indirect association can be provided by the association of omission of such treatments and the reported importance of coenurosis in replacement animals in the farm. Coenurosis has been declared the second most important health problem in replacement animals in Greece [21]. The acute form of cerebral coenurosis is more frequent in young animals [33,34], and the relevant clinical signs are considered to be the consequences of an acute diffuse inflammatory process resulting from toxic or hypersensitivity reactions taking place after infection [35].

## 5. Conclusions

This study provided a countrywide evaluation of the gastrointestinal parasitic infections in farm dogs. Hookworms and *T. canis* were found to be the most frequently detected helminths in dogs. An association was seen between the presence of canid predators near the farms and parasitic infections in the dogs.

The findings have also suggested the existence of interactions between the management of farm dogs and that of the livestock on the farms. It may be suggested that some farmers neglected both their livestock and farm dogs. This attitude may have influenced the health of all the animals on their farms. These interactions are reflected in the anthelmintic treatments of the animals and include (i) the financing of farm dog treatments from livestock-derived income, (ii) the apparently similar approaches in performing anthelmintic treatments in the animals (livestock and farm dogs) and (iii) the adverse effects of omitting anthelmintic treatments in farm dogs for the health of the livestock.

## Figures and Tables

**Table 1:** Results of multivariable analysis for parameters with significant association with presence of farm dogs in small ruminant farms in Greece.

Variables	Odds Ratio (95% CI)/ Odds Risk (±s.e.) ^1^	*p*
Presence of wild mammal predators near the farm		0.008
Yes (96.0% ^2^)	3.070 (1.418–6.648)	0.004
No (88.7%)	Reference	-
Daily period of farmers’ presence at the farm		0.021
Per-unit (h) increase (*r_sp_* = 0.097)	1.007 ± 1.003	0.031
Livestock species on the farm		0.044
Sheep (91.4% ^2^)	Reference	
Goats (96.6%)	2.710 (0.930–7.898)	0.07
Management system applied in the farm		0.046
Intensive or semi-intensive (95.0% ^2^)	2.004 (0.942–4.262)	0.07
Semi-extensive or extensive (90.5%)	Reference	

^1^ CI: confidence interval, se: standard error; ^2^ proportion of farms with the outcome under evaluation.

**Table 2:** Frequency of detection of eggs of gastrointestinal helminths in faecal samples from dogs in small ruminant farms in Greece.

Helminths	Frequency of Detection
Hookworms (*Uncinaria*/*Ancylostoma*)	155 (68.6% ^1^)
*Toxocara canis*	116 (51.3%)
*Toxascaris leonina*	59 (26.1%)
*Dipylidium caninum*	41 (18.1%)
*Capillaria* spp.	9 (4.0%)
*Trichuris vulpis*	8 (3.5%)
Taeniidae family (*Taenia* spp., *Echinococcus* spp.)	3 (1.3%)

^1^ proportion among farms from which faecal samples were collected from dogs.

**Table 3:** Results of multivariable analysis for parameters with significant association with detection of eggs of hookworms in faecal samples from farm dogs in small ruminant farms in Greece.

Variables	Odds Ratio (95% CI) ^1^	*p*
Presence of wild canid predators near the farm		0.008
Yes (75.7% ^2^)	2.281 (1.286–4.047)	0.005
No (57.8%)	Reference	-
Professional status of farmers		0.042
Full-time (66.7% ^2^)	Reference	-
Part-time (89.5%)	4.250 (0.955–18.922)	0.06

^1^ CI: confidence interval; ^2^ proportion of farms with outcome under evaluation.

**Table 4:** Results of multivariable analysis for parameters with significant association with detection of eggs of *T. canis* in faecal samples from farm dogs in small ruminant farms in Greece.

Variables	Odds Ratio (95% CI) ^1^	*p*
Presence of wild canid predators near the farm		0.004
Yes (59.6% ^2^)	2.314 (1.342–3.991)	0.003
No (38.9%)	Reference	-

^1^ CI: confidence interval; ^2^ proportion of farms with outcome under evaluation.

**Table 5:** Results of multivariable analysis for parameters with significant association with omission of anthelmintic administration to farm dogs.

Variables	Odds Ratio (95% CI)/ Odds Risk (±s.e.) ^1^	*p*
Management system applied in farms		0.004
Intensive or semi-intensive (10.0% ^2^)	Reference	-
Semi-extensive or extensive (22.4%)	2.610 (1.491–4.567)	0.0008
Annual milk production per animal		0.004
Per-unit (L) increase (*r_sp_* = 0.261)	1.001 ± 1.001	<0.0001
Collaboration with veterinary practice		0.020
Yes (14.0% ^2^)	Reference	-
No (31.3%)	2.790 (1.416–5.497)	0.003

^1^ CI: confidence interval, se: standard error; ^2^ proportion of farms with outcome under evaluation.

**Table 6:** Eigenvalues for principal component analysis for citations received by published papers yearly.

Parameter	PC_1_	PC_2_	PC_3_	PC_4_
Eigenvalue	1.53	1.03	0.83	0.61
% of variance	38.1	25.9	20.7	15.4
Cumulative variance (%)	38.1	64.0	84.6	100.0

**Table 7:** Frequency of use of anthelmintics or combinations of anthelmintics administered to farm dogs in Greece.

Combinations of Anthelmintic Drugs	No. of Farms Using the Combination
[Praziquantel, pyrantel, febantel] ^1^	313 (90.46% ^2^)
Ivermectin; milbemycin	19 (5.49%)
[Praziquantel, pyrantel, febantel]; ivermectin	6 (1.73%)
Ivermectin	3 (0.87%)
[Praziquantel, pyrantel, febantel]; [praziquantel, milbemycin]	2 (0.58%)
[Praziquantel, milbemycin]	2 (0.58%)
[Praziquantel]	1 (0.29%)

^1^ drugs within the square brackets indicate a product including the combination of these drugs; ^2^ proportion among farms in which the administration of anthelmintics to farm dogs was reported.

## Data Availability

Most of the data presented in this study are in Supplementary Materials. The remaining data are available upon request from the corresponding author. The data are not publicly available, as they form part of the PhD thesis of the first author, which has not yet been examined, approved, nor uploaded in the official depository of PhD theses from Greek universities.

## Supplementary Materials

The following supporting information can be downloaded at https://www.mdpi.com/article/10.3390/ani14223233/s1, Table S1: Information (related to infrastructure, animals, production characteristics, health management, human resources and climatic conditions) obtained for the study of gastrointestinal parasitic infection in farm dogs in 325 sheep flocks and 119 goat herds in Greece; Table S2: Details of the multivariable model employed for the evaluation of associations with the administration of anthelmintics to farm dogs in 412 small ruminant farms in Greece; Table S3: Results of univariable analyses for associations with the presence of farm dogs in small ruminant farms in Greece; Figure S1: Pictures of helminth eggs detected more frequently in faecal samples from farm dogs in small ruminant farms in Greece; Table S4: Results of univariable analyses for associations with the detection of the eggs of hookworms in faecal samples from farm dogs in small ruminant farms in Greece; Table S5: Results of univariable analyses for associations with the detection of the eggs of *Toxocara canis* in faecal samples from farm dogs in small ruminant farms in Greece; Table S6: Results of univariable analyses for associations with the omission of the administration of anthelmintics to farm dogs in small ruminant farms in Greece; Figure S2: Box and whisker plot of the age of small ruminant farmers, in accord with the omission (red plot) or performance (blue plot) of the administration of anthelmintics to the farm dogs; Table S7: Comparison of results of categorisation of farms (n) in accord with the administration of ivermectin to livestock and/or farm dogs.

## References

[1] van Bommel L., Magrath M., Coulson G., Johnson C.N. (2024). Livestock guardian dogs establish a landscape of fear for wild predators: Implications for the role of guardian dogs in reducing human-wildlife conflict and supporting biodiversity conservation. Ecol. Solut. Evid..

[2] Sepúlveda M.A., Singer R.S., Silva-Rodríguez E., Stowhas P., Pelican K. (2014). Domestic dogs in rural communities around protected areas: Conservation problem or conflict solution?. PLoS ONE.

[3] Ugarte C.S., Talbot-Wright T., Simonetti J.A. (2021). Two for the price of one: Livestock guarding dogs deter not only predators but also competitors. Rangel. Ecol. Manag..

[4] Okoh A.E.J. (1981). Rabies in farm livestock in Nigeria. Int. J. Zoonos..

[5] Salaberry S.R.S., Castro V., Nassar A.F.C., Castro J.R., Guimaraes E.C., Lima-Ribeiro A.M.C. (2011). *Leptospira* spp. in ovines from Uberlandia municipality, Minas Gerais state, Brazil. Braz. J. Microbiol..

[6] Rubel W., Schoneberg C., Wolf A., Ganter M., Bauer B.U. (2021). Seroprevalence and risk factors of *Anaplasma* spp. in German small ruminant flocks. Animals.

[7] Esmaeilnejad B., Tavassoli M., Asri-Rezaei S., Dalir-Naghadeh B., Mardani K., Golabi M., Arjmand J., Kazemnia A., Jalilzadeh G. (2015). Determination of prevalence and risk factors of infection with *Babesia ovis* in small ruminants from West Azerbaijan province, Iran by polymerase chain reaction. J. Arthropod. Borne Dis..

[8] Varcasia A., Tamponi C., Ahmed F., Cappai M.G., Porcu F., Mehmood N., Dessì G., Scala A. (2022). *Taenia multiceps* coenurosis: A review. Parasit. Vectors.

[9] Christodoulopoulos G., Theodoropoulos G., Petrakos G. (2008). Epidemiological survey of cestode-larva disease in Greek sheep flocks. Vet. Parasitol..

[10] Bush A.O., Fernandez J.C., Esch G.W., Seed J. (2001). Parasitism: The Diversity and Ecology of Animal Parasites.

[11] Aldawek A.M., Levkut M., Revajová V., Kolodzieyski L., Ševeiková Z., Dubinský P. (2002). Larval toxocarosis in sheep: The immunohistochemical characterization of lesions in some affected organs. Vet. Parasitol..

[12] Lianou D.T., Chatziprodromidou I.P., Vasileiou N.G.C., Michael C.K., Mavrogianni V.S., Politis A.P., Kordalis N.G., Billinis C., Giannakopoulos A., Papadopoulos E. (2020). A detailed questionnaire for the evaluation of health management in dairy sheep and goats. Animals.

[13] Bowman D.D. (2014). Georgis’ Parasitology for Veterinarians.

[14] Symeonidou I., Gelasakis A.I., Arsenopoulos K.V., Schaper R., Papadopoulos E. (2017). Regression models to assess the risk factors of canine gastrointestinal parasitism. Vet. Parasitol..

[15] Katsarou E.I., Lianou D.T., Papadopoulos E., Fthenakis G.C. (2022). Long-term climatic changes in small ruminant farms in Greece and potential associations with animal health. Sustainability.

[16] Gehring T.M., VerCauteren K.C., Landry J.M. (2010). Livestock protection dogs in the 21st century: Is an ancient tool relevant to modern conservation challenges?. BioScience.

[17] Ivaşcu C.M., Biro A. (2020). coexistence through the ages: The role of native livestock guardian dogs and traditional ecological knowledge as key resources in conflict mitigation between pastoralists and large carnivores in the Romanian Carpathians. J. Ethnobiol..

[18] Kennel Club of Greece Greek Sheepdog (Hellinikos Pimenikos). https://www.koe.gr/index.php/en/greek-breeds/greek-sheepdog-hellinikos-pimenikos.

[19] Yilmaz O., Erturk Y.E., Coskun F., Ertugrul M. (2015). Using livestock guardian dogs in Balkans. Agric. Forest..

[20] Cozza K., Fico R., Battistini M.L., Rogers E. (2016). The damage–conservation interface illustrated by predation on domestic livestock in central Italy. Biol. Conserv..

[21] Lianou D.T., Michael C.K., Fthenakis G.C. (2023). Data on mapping 444 dairy small ruminant farms during a countrywide investigation performed in Greece. Animals.

[22] Arcturos Greek Sheepherd Dogs. https://www.arcturos.gr/en/activities/greek-sheperd-dogs/.

[23] Gonzàlez A., Navaro A., Funes M., Pailacura O., Bolgeri M.J., Walker S. (2012). Mixed-breed guarding dogs reduce conflict between goat herders and native carnivores in Patagonia. Hum.-Wildl. Interact..

[24] Rust A., Whitehouse-Tedd M., MacMillan D. (2013). Perceived efficacy of livestock-guarding dogs in South Africa: Implications for cheetah conservation. Wildl. Soc. Bull..

[25] Kloch A., Bednarska M., Bajer A. (2005). Intestinal macro- and microparasites of wolves (*Canis lupus* L.) from north-eastern Poland recovered by coprological study. Ann. Agric. Environ. Med..

[26] Balinsky D.L., Paras K.L., Hanna R., Elsemore D.A., Verocai G.G. (2019). Parasite survey on a captive wolf population using classical techniques and ELISA coproantigen detection, USA. Vet. Parasitol. Reg. Stud. Rep..

[27] Tsokana C.N., Sioutas G., Symeonidou I., Papadopoulos E. (2024). Wildlife and parasitic infections: A One Health perspective in Greece. Curr. Res. Parasitol. Vector Borne Dis..

[28] Papavasili T.E., Kontogeorgos A., Mayrommati A., Chatzitheodoridis F., Sossidou E.N. (2023). Defining priority issues for managing stray dog populations: The case of Greece. J. Hell. Vet. Med. Soc..

[29] Petaliou S. Greece Has Been Overwhelmed with Stray Dogs. https://www.newsbeast.gr/environment/arthro/9256928/giati-i-ellada-echei-gemisei-me-adespota-skylia-70-000-zoun-egkataleleimmena-sta-vouna-kai-3-ekat-se-oli-ti-chora.

[30] Corner-Thomas R.A., Kenyon P.R., Morris S.T., Ridler A.L., Hickson R.E., Greer A.W., Logan C.M., Blair H.T. (2015). Influence of demographic factors on the use of farm management tools by New Zealand farmers. N. Z. J. Agric. Res..

[31] Marelli S.P., Polli M., Frattini S., Cortellari M., Rizzi R., Crepaldi P. (2020). Genotypic and allelic frequencies of MDR1 gene in dogs in Italy. Vet. Rec. Open.

[32] Barroso M.C., Grilo A., Aguiar S., Aires da Silva F., São Braz B. (2022). Occurrence of MDR1 1-delta mutation in herding dog breeds in Portugal. Front. Vet. Sci..

[33] Dyson D.A., Linklater K.A. (1979). Problems in the diagnosis of coenurosis in sheep. Vet. Rec..

[34] Giadinis N.D., Psychas V., Polizopoulou Z., Papadopoulos E., Papaioannou N., Komnenou A.T., Thomas A.L., Petridou E.J., Kritsepi-Konstantinou M., Lafi S.Q. (2012). Acute coenurosis of dairy sheep from 11 flocks in Greece. N. Z. Vet. J..

[35] Edwards G.T., Herbert I.V. (1982). Observations on the course of *Taenia multiceps* infections in sheep: Clinical signs and post-mortem findings. Br. Vet. J..

