# Supplementary material for: Gastrointestinal Helminth Infections in Dogs in Sheep and Goat Farms in Greece: Prevalence, Involvement of Wild Canid Predators and Use of Anthelmintics
Source: Animals (Basel). 2024 Nov 12;14(22):3233. doi: 10.3390/ani14223233 (PMC11591064; doi:10.3390/ani14223233)
Supplement: Supplementary file 1 [file animals-14-03233-s001.zip › animals-3299705-supplementary.pdf]

Gastrointestinal Helminth Infections in Dogs in Sheep and Goat Farms in Greece: Prevalence, Involvement of Wild Canid Predators and Use of Anthelmintics

Eleni I. Katsarou [1], Konstantinos V. Arsenopoulos [2,†], Charalambia K. Michael [3,†], Daphne T. Lianou [1], Efthymia Petinaki [4], Elias Papadopoulos [2] and George C. Fthenakis [1,*]

Table S1. Information (related to infrastructure, animals, production characteristics, health management, human resources and climatic conditions) obtained for the study of gastrointestinal parasitic infection in farms dogs in 325 sheep flocks and 119 goat herds in Greece.

Variables related to sampling conditions
Season of sampling (description)
Variables related to infrastructure in farms
Management system applied in farm (EFSA classification: shepherding / intensive / semi-intensive / semi-extensive / extensive / very extensive / mixed)
Altitude at the location of farm (m)
Variables related to animals on the farm
Livestock species on the farm (sheep / goats)
No. of adult livestock animals on farms (no.)
Presence of dogs on farm (yes / no)
Number of dogs on farm (no.)
Visual contacts of the farmer with wildlife mammals (yes / no)
Wildlife mammals identified within a radius of 2 km of the farm (description)
Variables related to production characteristicis in farms
Total milk quantity obtained during the preceding milking period (litres)
Number of lambs / kids born during the preceding lambing / kidding season (no.)
Variables related to health management in farms
Duration of grazing during the winter (no. of months)
Duration of grazing during the summer (no. of months)
Collaboration with a veterinarian (yes / no)
Annual frequency of systemic disinfections in the farm (no. of occasions)
Seasonal transfer of animals to other site (transhumance) (yes / no)
Disposal of carcasses from dead livestock (incineration / burying / feeding to dogs / feeding to birds / drop-off away)
Administration of anthelmintics to sheep / goats in the farm (yes / no)
Anthelmintics administered (description)
Administration of antiparasitics to dogs on the farm (yes / no)
Antiparasitics used (description)
Variables related to human resources in farms
Age of farmer (years)
Length of previous animal farming experience (years)

Highest general education level achieved (primary / secondary / tertiary)
Farmer by profession (yes / no)
Daily period of presence at the farm (hours)
Family tradition in farming (yes / no)
Total members of the family (no.)
Presence of working staff at the farm (yes / no)
Hunting activity by the farmer (yes / no)
Variables related to climatic conditions at the locations of farms
Temperature at 2 m for the three months prior to the visit (°C)
Temperature of Earth skin for the three months prior to the visit (°C)
Minimum temperature at 2 m for the three months prior to the visit (°C)
Maximum temperature at 2 m for the three months prior to the visit (°C)
Temperature range at 2 m for the three months prior to the visit (°C)
Relative humidity at 2 m for the three months prior to the visit (%)
Precipitation for the three months prior to the visit (kg m^{-2} s^{-1})
Temperature at 2 m for the year preceding the visit (°C)
Temperature of Earth skin for the year preceding the visit (°C)
Minimum temperature at 2 m for the year preceding the visit (°C)
Maximum temperature at 2 m for the year preceding the visit (°C)
Temperature range at 2 m for the year preceding the visit (°C)
Relative humidity at 2 m for the year preceding the visit (%)
Precipitation for the year preceding the visit (kg m^{-2} s^{-1})
Wind speed at 10 m for the year preceding the visit (m s^{-1})

Table S2. Details of the multivariable model employed for the evaluation of associations with the administration of anthelmintics to farm dogs in 412 small ruminant farms in Greece.

Outcome	Variables		
	assessed in univariable analyses (*n*)	offered to the multi-variable models (*n*)	required in the final models
Presence of farm dogs in small ruminant farms	16	6	(a) management system applied in the farm, (b) livestock species on the farm, (c) presence of wild mammal predators near the farm, (d) length of previous animal farming experience, (e) daily period of farmer's presence at the farm
Detection of hookwarms in faecal samples from a farm	34	7	(a) presence of wild mammal predators near the farm, (b) annual frequency of systemic disinfections in the farm, (c) farmer by profession, (d) presence of working staff at the farm
Detection of *T. canis* in faecal samples from a farm	34	4	(a) season of sampling, (b) presence of wild mammal predators near the farm, (c) disposal of carcasses from dead livestock, (d) farmer by profession
Omission of the administration of anthelmintics to farm dogs	16	9	(a) management system applied in the farm, (b) duration of grazing of the livestock annually, (c) annual milk production per ewe / doe, (d) collaboration with a veterinarian, (e) age of farmer, (f) farmer by profession

Table S3. Results of univariable analyses for associations with the presence of farm dogs in small ruminant farms in Greece.

Farms where no farm dogs were present (n = 32)			Farms where farm dogs were present (n = 412)			p
Management system applied in farm						
intensive or semi-intensive	semi-extensive or extensive		intensive or semi-intensive	semi-extensive or extensive		
11	21		211	202		0.007
Altitude at the location of farm						
152.5 (202.5) m			167.5 (323.5) m			0.82
Livestock species on the farm						
Sheep	Goats		Sheep	Goats		
28	4		297	115		0.06
No. of adult livestock animals in farm						
219 (271) animals			240 (255) animals			0.88
Presence of wild mammal predators near the farm						
Yes	No		Yes	No		
10	22		240	172		0.003
Presence of wild mammal meso-predators near the farm						
Yes	No		Yes	No		
27	5		328	84		0.52
Average milk quantity obtained per animal during the preceding milking period						
200.0 (137.5) L			189.5 (129.5) L			0.62
Average number of lambs / kids born per animal during the preceding lambing / kidding season						
1.3 (0.3)			1.3 (0.2)			0.73
Annual duration of grazing						
8.5 (6.5) months			8.0 (6.5) months			0.61
Age of farmer						
50.5 (16.5) years			46.5 (18.0) years			0.17
Length of previous animal farming experience						
30.0 (22.5) years			25.0 (25.0) years			0.07
Highest general education level achieved						
Primary	Secondary	Tertiary	Primary	Secondary	Tertiary	
5	24	3	72	290	50	0.84
Farmer by profession						
Yes	No		Yes	No		
28	4		369	43		0.71
Daily period of presence at the farm						
10.0 (7.0) hours			15.0 (7.0) hours			0.042

Family tradition in farming				
Yes	No	Yes	No	
28	4	358	54	0.92
Presence of working staff at the farm				
Yes	No	Yes	No	
9	23	148	264	0.37

Figure S1. Pictures of helminth eggs deetcted more frequently in faecal samples from farm dogs in small ruminant farms in Greece (from left to right: hookworm (*Uncinaria* / *Ancylostoma*) egg, *Toxocara canis* eggs, *Toxascaris leonina* eggs, *Dipylidium caninum* eggs).

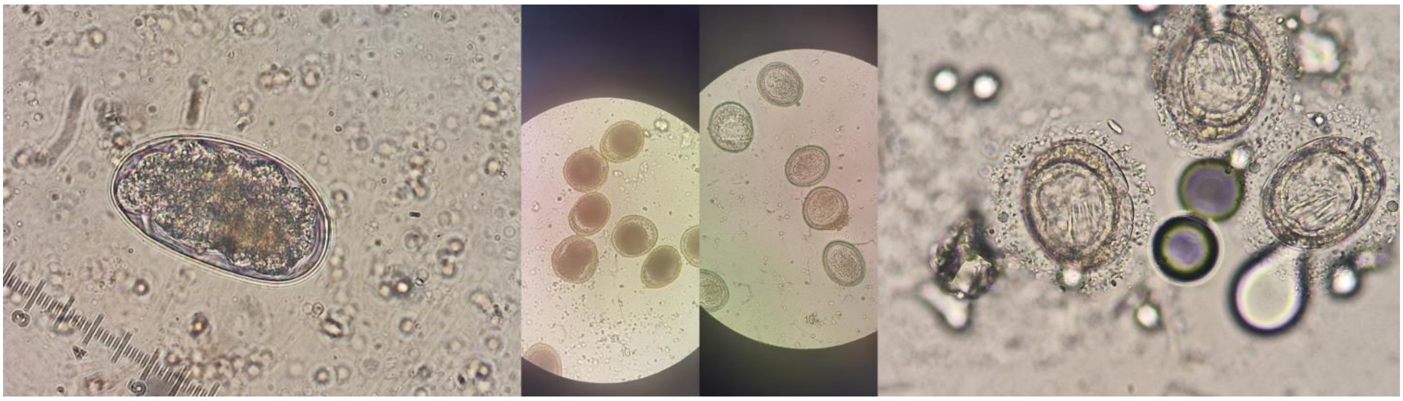

Table S4. Results of univariable analyses for associations with the detection of eggs of hookworms in faecal samples from farm dogs in small ruminant farms in Greece.

Farms where no eggs of hookworms were detected (n = 71)			Farms where eggs of hookworms were detected (n = 155)			p
Season of sampling						
Spring	Summer	Winter	Spring	Summer	Winter	
21	28	22	79	46	30	0.009
Management system applied in farm						
intensive or semi-intensive	semi-extensive or extensive		intensive or semi-intensive	semi-extensive or extensive		
33	38		85	70		0.24
Altitude at the location of farm						
118.0 (496.5) m			177.0 (314.5)			0.74
Livestock species on the farm						
Sheep	Goats		Sheep	Goats		
47	24		101	54		0.88
Number of dogs on farm						
5 (7)			4 (6)			0.77
Presence of wild mammal predators near the farm						
Yes	No		Yes	No		
33	38		103	52		0.004
Annual duration of grazing						
9 (6) months			8 (7) months			0.32
Collaboration with a veterinarian						
Yes	No		Yes	No		
62	9		140	15		0.50
Annual frequency of systemic disinfections in the farm						
2 (3) occasions			2 (3) occasions			0.10
Seasonal transfer of animals to other site						
Yes	No		Yes	No		
14	57		28	127		0.77
Disposal of carcasses from dead livestock animals						
incineration / burying	feeding to dogs / feeding to birds / drop-off away		incineration / burying	feeding to dogs / feeding to birds / drop-off away		
27	44		75	60		0.14
Administration of anthelmintics to dogs on the farm						
Yes	No		Yes	No		
54	17		120	30		0.50

Age of farmer						
	49.0 (18.5) years			45.0 (17.0) years		0.025
Length of previous animal farming experience						
	25.0 (22.5) years			25.0 (12.5) years		0.81
Highest general education level achieved						
Primary	Secondary	Tertiary	Primary	Secondary	Tertiary	
12	52	7	25	106	24	0.52
Farmer by profession						
Yes		No	Yes		No	
69		2	138		17	0.040
Daily period of presence at the farm						
	15.0 (7.0) hours			15.0 (7.0) hours		0.46
Family tradition in farming						
Yes		No	Yes		No	
60		11	134		21	0.70
Presence of working staff at the farm						
Yes		No	Yes		No	
30		41	49		106	0.12
Hunting activity by the farm						
Yes		No	Yes		No	
11		60	26		169	0.81
Temperature at 2 m for the three months prior to the visit						
	15.8 (3.0) °C			15.8 (1.1) °C		0.60
Temperature of Earth skin for the three months prior to the visit						
	16.0 (4.4) °C			16.0 (1.5) °C		0.50
Minimum temperature at 2 m for the three months prior to the visit						
	–3.1 (7.0) °C			–3.1 (3.4) °C		0.86
Maximum temperature at 2 m for the three months prior to the visit						
	35.3 (3.9) °C			35.6 (3.5) °C		0.85
Temperature range at 2 m for the three months prior to the visit						
	38.4 (7.0) °C			38.7 (5.4) °C		0.45
Relative humidity at 2 m for the three months prior to the visit						
	69.7% (1.6%)			69.7% (1.3%)		0.79
Precipitation for the three months prior to the visit						
	1.9 (0.5) kg m^{-2} s^{-1}			1.9 (0.5) kg m^{-2} s^{-1}		0.54
Temperature at 2 m for the year prior to the visit						
	16.0 (2.5) °C			16.0 (1.1) °C		0.44

Temperature of Earth skin for the year prior to the visit		
16.3 (3.2) °C	16.1 (1.5) °C	0.49
Minimum temperature at 2 m for the year prior to the visit		
–5.3 (9.6) °C	–5.6 (4.5) °C	0.28
Maximum temperature at 2 m for the year prior to the visit		
37.9 (3.9) °C	38.0 (3.3) °C	0.68
Temperature range at 2 m for the year prior to the visit		
42.3 (11.6) °C	43.7 (7.5) °C	0.34
Relative humidity at 2 m for the year prior to the visit		
68.3% (5.3%)	68.3% (2.4%)	0.98
Precipitation for the year prior to the visit		
2.0 (0.55) kg m^{-2} s^{-1}	1.9 (0.7) kg m^{-2} s^{-1}	0.72

Table S5. Results of univariable analyses for associations with the detection of eggs of *Toxocara canis* in faecal samples from farm dogs in small ruminant farms in Greece.

Farms where no eggs of *T. canis* were detected (n = 110)		Farms where eggs of *T. canis* were detected (n = 116)		p
Season of sampling				
Spring / Summer / Winter		Spring / Summer / Winter		
43 / 36 / 31		57 / 38 / 21		0.15
Management system applied in farm				
intensive or semi-intensive	semi-extensive or extensive	intensive or semi-intensive	semi-extensive or extensive	
61	49	57	59	0.34
Altitude at the location of farm				
137.5 (256.5) m		178.0 (356.0)		0.33
Livestock species on the farm				
Sheep	Goats	Sheep	Goats	
76	34	72	44	0.27
Number of dogs on farm				
5 (7)		4 (7)		0.47
Presence of wild mammal predators near the farm				
Yes	No	Yes	No	
55	55	81	35	0.002
Annual duration of grazing				
9 (6) months		8 (7) months		0.32
Collaboration with a veterinarian				
Yes	No	Yes	No	
101	9	101	15	0.25
Annual frequency of systemic disinfections in the farm				
2 (2) occasions		2 (3) occasions		0.21
Seasonal transfer of animals to other site				
Yes	No	Yes	No	
19	91	23	93	0.62
Disposal of carcasses from dead livestock animals				
incineration / burying	feeding to dogs / feeding to birds / drop-off away	incineration / burying	feeding to dogs / feeding to birds / drop-off away	
44	66	58	58	0.13
Administration of anthelmintics to dogs on the farm				
Yes	No	Yes	No	
86	24	88	28	0.68

Age of farmer						
	47.0 (16.0) years			46.0 (18.0) years		0.24
Length of previous animal farming experience						
	25.0 (20.0) years			25.0 (16.3) years		0.61
Highest general education level achieved						
Primary	Secondary	Tertiary	Primary	Secondary	Tertiary	
13	82	15	24	76	16	0.19
Farmer by profession						
Yes		No	Yes		No	
105		5	102		14	0.042
Daily period of presence at the farm						
	15.0 (6.5) hours			10.0 (7.0) hours		0.28
Family tradition in farming						
Yes		No	Yes		No	
91		19	103		13	0.19
Presence of working staff at the farm						
Yes		No	Yes		No	
45		65	34		82	0.07
Hunting activity by the farm						
Yes		No	Yes		No	
15		95	22		94	0.28
Temperature at 2 m for the three months prior to the visit						
	15.8 (3.0) °C			15.8 (1.1) °C		0.86
Temperature of Earth skin for the three months prior to the visit						
	16.0 (4.0) °C			16.0 (1.4) °C		0.90
Minimum temperature at 2 m for the three months prior to the visit						
	–3.1 (6.7) °C			–3.1 (3.3) °C		0.63
Maximum temperature at 2 m for the three months prior to the visit						
	35.4 (3.6) °C			35.6 (3.5) °C		0.50
Temperature range at 2 m for the three months prior to the visit						
	38.4 (7.0) °C			38.7 (5.4) °C		0.21
Relative humidity at 2 m for the three months prior to the visit						
	69.7% (1.5%)			69.7% (1.8%)		0.81
Precipitation for the three months prior to the visit						
	1.9 (0.5) kg m^{-2} s^{-1}			1.9 (0.5) kg m^{-2} s^{-1}		0.87
Temperature at 2 m for the year prior to the visit						
	15.8 (2.5) °C			16.0 (1.1) °C		0.78

Temperature of Earth skin for the year prior to the visit		
16.1 (3.2) °C	16.2 (1.5)	0.74
Minimum temperature at 2 m for the year prior to the visit		
−5.3 (9.2) °C	−5.3 (4.5) °C	0.58
Maximum temperature at 2 m for the year prior to the visit		
37.9 (4.2) °C	38.0 (2.6) °C	0.54
Temperature range at 2 m for the year prior to the visit		
42.5 (10.5) °C	43.9 (7.1) °C	0.38
Relative humidity at 2 m for the year prior to the visit		
68.1% (4.6%)	68.3% (2.9%)	0.76
Precipitation for the year prior to the visit		
2.0 (0.5) kg m^{-2} s^{-1}	1.9 (0.8) kg m^{-2} s^{-1}	0.47

Table S6. Results of univariable analyses for associations with the omission of the administration of anthelmintics to farm dogs in small ruminant farms in Greece.

Farms where anthelmintic treatment was omitted (n = 66)			Farms where anthelmintic treatment was not omitted (n = 346)			p
Management system applied in farm						
intensive or semi-intensive	semi-extensive or extensive		intensive or semi-intensive	semi-extensive or extensive		
21	45		190	56		0.0006
Altitude at the location of farm						
161.0 (260.5) m			168.0 (329.3)			0.39
Livestock species on the farm						
Sheep	Goats		Sheep	Goats		
43	23		254	98		0.25
No. of adult livestock animals on farms						
274 (230)			238 (260)			0.34
Average milk quantity obtained per animal during the preceding milking period						
127.3 (82.1) L			200.0 (126.0) L			< 0.0001
Average number of lambs / kids born per animal during the preceding lambing / kidding season						
1.2 (0.2)			1.3 (0.2)			0.006
Annual duration of grazing						
11 (6) months			8 (7) months			0.035
Collaboration with a veterinarian						
Yes	No		Yes	No		
51	16		313	33		0.0009
Age of farmer						
53.0 (17.8) years			45.5 (18.0) years			0.022
Length of previous animal farming experience						
25.0 (28.3) years			25.0 (25.0) years			0.34
Highest general education level achieved						
Primary	Secondary	Tertiary	Primary	Secondary	Tertiary	
11	50	5	61	240	45	0.43
Farmer by profession						
Yes	No		Yes	No		
64	2		305	41		0.032
Daily period of presence at the farm						
15.0 (5.0) hours			15.0 (7.0) hours			0.71

Family tradition in farming				
Yes	No	Yes	No	
63	3	296	51	0.025
Presence of working staff at the farm				
Yes	No	Yes	No	
19	47	130	216	0.17
Total members of the family				
4 (2)		4 (2)		0.64

Figure S2. Box and whisker plot of the age of small ruminant farmers, in accord with the omission (red plot) or performance (blue plot) of administration of anthelmintics to the farm dogs.

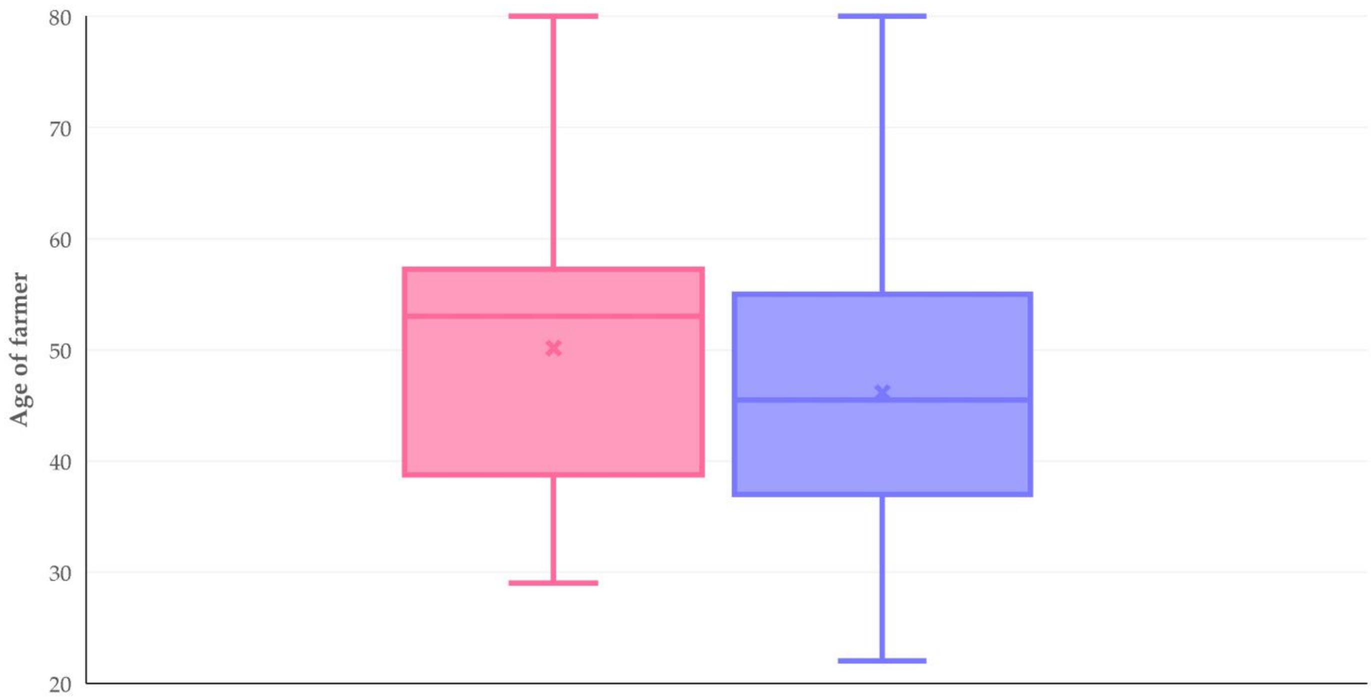

Table S7. Comparison of results of categorization of farms (*n*) in accord with the administration of ivermectin to livestock and / or farm dogs.

		Administration of ivermectin to livestock	
		Yes	No
Administration of ivermectin to farm dogs	Yes	26	2
	No	202	182
